# LOX is a novel mitotic spindle-associated protein essential for mitosis

**DOI:** 10.18632/oncotarget.8628

**Published:** 2016-04-07

**Authors:** Myriem Boufraqech, Darmood Wei, Urbain Weyemi, Lisa Zhang, Martha Quezado, Petr Kalab, Electron Kebebew

**Affiliations:** ^1^ Endocrine Oncology Branch, Center for Cancer Research, National Cancer Institute, National Institutes of Health, Bethesda, MD 20892, USA; ^2^ Urology Oncology Branch, Center for Cancer Research, National Cancer Institute, National Institutes of Health, Bethesda, MD 20892, USA; ^3^ Laboratory of Molecular Pharmacology, Center for Cancer Research, National Cancer Institute, National Institutes of Health, Bethesda, MD 20892, USA; ^4^ Laboratory of Pathology, Center for Cancer Research, National Cancer Institute, National Institutes of Health, Bethesda, MD 20892, USA; ^5^ Laboratory of Cellular and Molecular Biology, Center for Cancer Research, National Cancer Institute, National Institutes of Health, Bethesda, MD 20892, USA

**Keywords:** LOX, microtubules, cell cycle, mitosis, cancer

## Abstract

*LOX* regulates cancer progression in a variety of human malignancies. It is overexpressed in aggressive cancers and higher expression of LOX is associated with higher cancer mortality. Here, we report a new function of LOX in mitosis. We show that LOX co-localizes to mitotic spindles from metaphase to telophase, and p-H3^(Ser10)^-positive cells harbor strong LOX staining. Further, purification of mitotic spindles from synchronized cells show that LOX fails to bind to microtubules in the presence of nocodazole, whereas paclitaxel treated samples showed enrichment in LOX expression, suggesting that LOX binds to stabilized microtubules. *LOX* knockdown leads to G2/M phase arrest; reduced p-H3^(Ser10)^, cyclin B1, CDK1, and Aurora B. Moreover, LOX knockdown significantly increased sensitivity of cancer cells to chemotherapeutic agents that target microtubules. Our findings suggest that LOX has a role in cancer cell mitosis and may be targeted to enhance the activity of microtubule inhibitors for cancer therapy.

## INTRODUCTION

Mitosis is a critical cell cycle phase, and its precise orchestration is necessary for the maintenance of chromosomal stability in cells [1]. Several important proteins that coordinate the spindle formation and the chromosomes’ dynamics during mitosis have been discovered. The key proteins involved in G_2_/M transition, mitotic entry, mitotic spindle assembly, chromatin condensation and segregation, and the cleavage furrow and midbody during cytokinesis consist of CDC25C, CDK1, CDK 2, Aurora A (AURKA) and B (AURKB), and polo-like kinase 1 (PLK1) [2, 3]. Current cancer therapy focuses mainly on identifying novel targets crucial in cancer initiation and/or progression, such as proteins that coordinate several functions in the cell cycle. Targeting mitosis in cancer cells is widely exploited as a therapeutic strategy. Drugs that selectively inhibit mitotic progression by disrupting spindles and kinetochore functions, and restricting key mitotic regulatory proteins are currently in various stages of clinical trials. Inhibition of the main regulators of mitotic entry leads to either an arrest in G_2_ or prevents mitotic entry, or leads to cell death in mitosis, also known as mitotic catastrophe as a consequence of failure to complete mitosis [4, 5].

The lysyl oxidase (LOX) family has 5 members; LOX and LOX-like proteins (LOXL-1 to 4). All members of the LOX family are secreted, copper-dependent amine oxidase which play a critical role in the biogenesis of connective tissue matrices by cross-linking the extracellular matrix proteins, collagen and elastin [6–9]. *LOX* has been found to be upregulated in metastatic breast cancer, and higher expression of LOX is associated with shorter metastasis-free survival [10]. We recently discovered that LOX was involved in anaplastic thyroid cancer (ATC) progression and metastasis, and higher expression of LOX was associated with lower survival rates in patients with differentiated thyroid cancer [11]. One of the hallmarks of metastatic and aggressive cancers is their enhanced mitotic capacity. This, in fact, is one of the most important prognostic factor for many solid malignancies, which is measured by the number of mitotic cells per high-power field seen on histology. Given that we previously observed uniformly high expression of*LOX* in undifferentiated and poorly differentiated thyroid cancer, which are characterized by high mitotic count, we hypothesized that LOX may have a role in cancer cell mitosis [12]. In this study, we investigated the function and localization of LOX in mitosis.

## RESULTS

### LOX is highly expressed in mitotic cells, and colocalizes and binds to microtubules in mitotic cells

**Figure 1:**
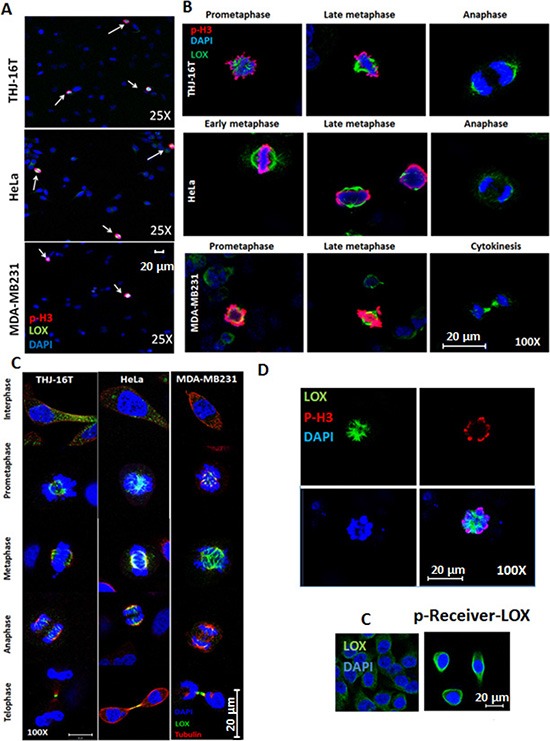
LOX is highly expressed in mitotic cells (**A**) LOX is highly expressed in p-H3^(Ser10)^-positive THJ-16T, HeLa, and MDA-MB231 cells (magnification 25X). (**B**) Subcellular localization of LOX in mitotic cells from prometaphase to anaphase (magnification 100X). (**C**) Co-localization of LOX and alpha tubulin on the mitotic spindles from metaphase to telophase (magnification 100X). (**D**) Top panel: p-H3^(Ser10)^ and LOX staining in LOX-HeLa cells (magnification 120X). Bottom panel: Representative images of HeLa cells transfected with p-Receiver-LOX vector compared to the control cells (magnification 25X). C: Control cells.

Immunofluorescence staining of LOX showed high expression of LOX in mitotic cells (p-H3-positive) (Figure 1A). We investigated the exact localization of LOX during mitotic progression. During pro-metaphase through anaphase, we observed a discernable enrichment of LOX by confocal microscopy in the spindle region in p-H3^(ser10)^-positive THJ-16T, MDA–MB231, and HeLa cells (Figure 1B). Co-immunostaining of LOX and alpha-tubulin showed colocalization of LOX on the mitotic spindles from metaphase to telophase (Figure 1C). To further determine whether LOX is also associated with mitotic spindles, we performed a transient transfection of a LOX-expressing vector in HeLa cells. We found strong LOX protein expression in p-H3 positive cells (Figure 1D). Taken together our data suggest the presence of LOX in the mitotic spindles during mitosis.

**Figure 2:**
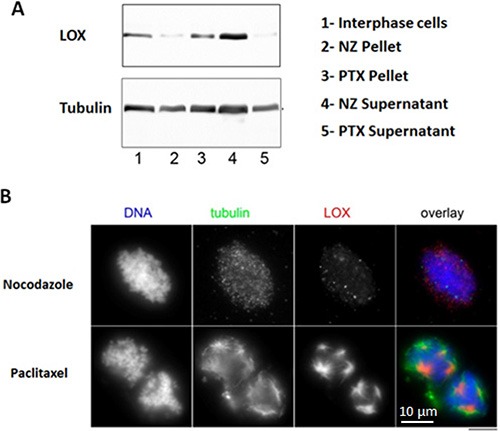
LOX binds to stabilized mitotic spindles (**A**) Stabilized microtubules with paclitaxel show enrichment in LOX expression, as compared with nonstabilized microtubules treated with nocodazole. (**B**) Co-localization of LOX and tubulin in paclitaxel-treated microtubules compared with nocodazole-treated samples. NZ: Nocodazole, PTX: Paclitaxel.

We next asked whether LOX interacts with mitotic microtubules because of its localization in mitotic spindles. To test this, we purified mitotic spindles from mitotically-synchronized HeLa cells and treated with either paclitaxel, which stabilizes polymerized microtubules, or nocodazole, which interferes with the microtubules polymerization. LOX failed to associate with the chromosomal pellets in the presence of nocodazole, whereas a significant amount of LOX co-pelleted with microtubules in paclitaxel-treated samples by Western blot (Figure 2A). Interestingly, immunofluorescence staining showed that while LOX strongly co-localized with dominant astral microtubules, it was absent or weaker in cytoplasmic microtubules that were disconnected from chromosomes (Figure 2B). This data indicates that the concentration of LOX in mitotic spindles is strongly associated with its microtubules-binding ability, and that this may be specific to the establishment of centrosomally-stabilized microtubules.

**Figure 3:**
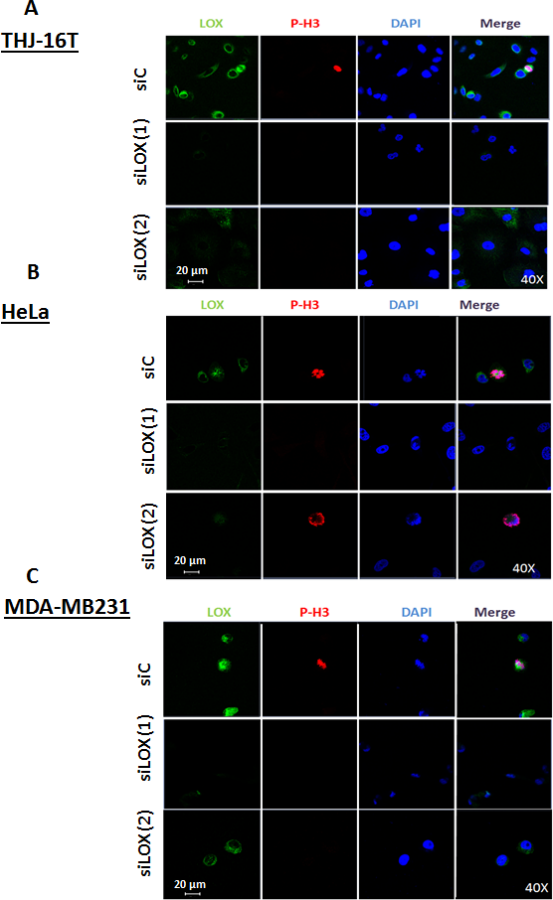
Knockdown of LOX protein using two independents siRNAs Immunofluorescence staining shows a decrease of LOX expression in siLOX (1) and siLOX (2) cells as compared to siControl in 3 cancer cells lines; THJ-16T (**A**), HeLa (**B**) and MDA-MB231 (**C**).

To confirm whether the interaction between LOX and the mitotic spindles is specific, cells were treated with 2 different small interference RNAs (siRNAs) targeting LOX expression or with scrambled siRNA (siC); and then immunostained with anti-LOX and anti-p-H3^(Ser10)^ antibodies. Confocal microscopy showed a significant decrease in LOX expression with both siLOX (1) and siLOX (2) cells compared to control cells (Figure 3A, 3B, 3C). However, siLOX (1) showed greater silencing potential than siLOX (2), and was therefore used for further analyses.

### Knockdown of LOX leads to accumulation of cells in the G_2_/M phase

**Figure 4:**
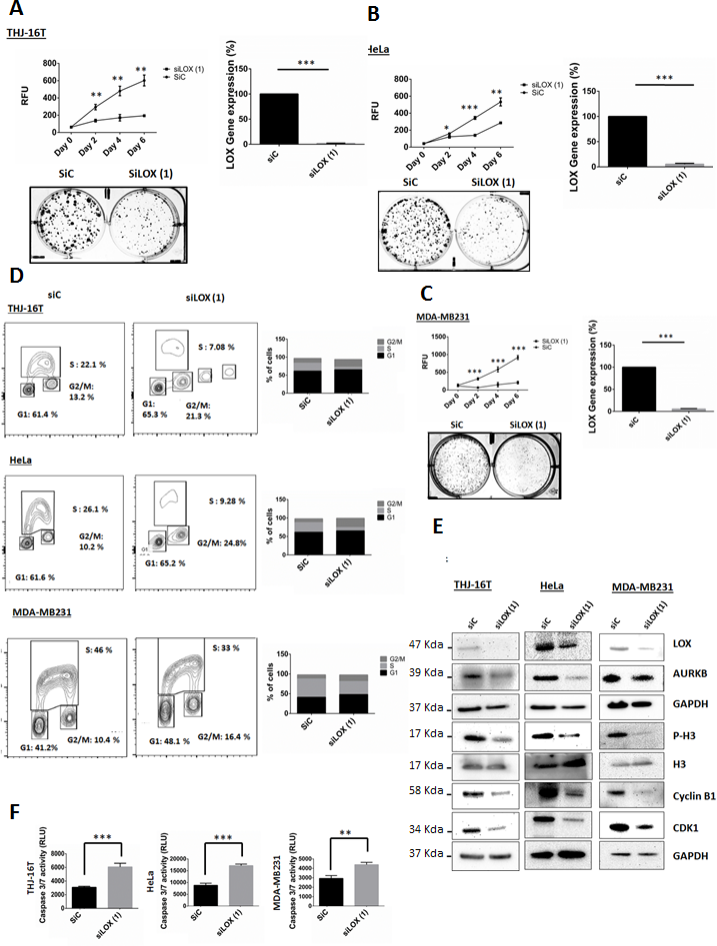
Knockdown of LOX affects cell proliferation, cell cycle progression and leads to apoptosis (**A**, **B**, **C**) Knockdown of LOX inhibits cellular proliferation and colonies formation in THJ-16T, HeLa and MDA-MB231 cells. (**D**) Cell cycle analysis using BrdU binding assay shows that LOX knockdown increases the number of cells in G2/M phase and decreases the number of cells in S phase. THJ-16T, HeLa, and MDA-MB231 cells were labeled for 2 hr with BrdU 72 hr after transfection with siLOX or its corresponding control. (**E**) Effects of LOX inhibition on G2/M transition markers. LOX knockdown decreased p-H3^(Ser10)^, cyclin B1, CDK1, and AUKB expression, 72 hr post-transfection. (**F**) Knockdown of LOX induces caspase-dependent apoptosis 72 hr after transfection. *p < 0.05, **p < 0.01, ***p < 0.001.

To evaluate whether LOX is required for the proliferation of cancer cells, we transfected cells with siLOX (1) or siLOX (2). Knockdown of LOX reduced the viability of the 3 cancer cell lines with a greater effect with siLOX (1) (Figure 4A, 4B, 4C and Supplementary Figure 1). To study the effects of LOX downregulation on long-term cell survival, we performed clonogenic assays in the cell lines. LOX knockdown decreased colony formation compared to control (Figure 4A, 4B, 4C and Supplementary Figure 1).

A significant increase in the percentage of cells in the G_2_/M phase and decrease in the percentage of cells in S phase were observed 72 hr after *LOX* knockdown (Figure 4D). The G_2_/M fraction increased by 1.6-, 2.4-, and 1.6-fold in THJ-16T, HeLa, and MDA-MB231 cells, respectively, as compared with control cells. To investigate the functional consequences of LOX repression on cell cycle progression, we used siRNA to knockdown its expression and BAPN to inhibit its enzymatic activity. We then analyzed several key proteins that are necessary for G_2_ to M transition phase. Histone 3^(Ser10)^ is phosphorylated in association with mitotic chromatin condensation in the late G_2_ and M phases of the cell cycle [13, 14]. This phosphorylation is mediated by AURKB [15], which ensures the proper microtubule-kinetochore attachment and normal separation of chromatid sisters [16–18]. On the other hand, active CyclinB1/CDK1 initially detected on centrosomes before transition into late G_2_ phase or in prophase [19], is phosphorylated on its cytoplasmic domain, leading subsequently to its nuclear translocation. This further enhances chromosome condensation and nuclear envelope breakdown [20]. High cyclin B1/CDK1 activity allows cells to stay in mitosis and all chromosomes to attach to the mitotic spindle. Progressive loss of cyclin B1/CDK1 activity is essential for successful chromosome segregation and completion of cell division [1]. The CDKs are negatively regulated by endogenous inhibitors, CDKIs, and p21 has been described to inhibit cyclin B1/CDK1 complexes, leading further to G_2_/M arrest.

In all three cancer cell lines, Western blot analysis showed a decrease of p-H3, cyclin B1, and AURKB, 72 hr after LOX knockdown, but not with BAPN treatment (Figures 4E and Supplementary Figure 2). Taken together, our data suggest that high endogenous levels of LOX help cells progress through mitosis, and that the mitotic entry is inhibited with the knockdown of LOX. On the other hand, inhibition of the enzymatic activity of LOX using BAPN did not affect either the cell cycle progression or the levels of p-H3 and cyclin B1 (Supplementary Figure 2). These data are consistent with a previous study showing that BAPN has no effect on cell proliferation or apoptosis [21, 22]. Thus, these observations suggest that the role of LOX in G_2_/M transition is independent of its catalytic activity.

Next, the cell lines were transfected with siLOX or siControl to investigate whether LOX knockdown induces caspase dependent apoptosis. The knockdown of LOX resulted in a marked increase of Caspase 3/7 activity 72 hr post-transfection. These results revealed that the G2/M phase arrest due to LOX depletion is accompanied by caspase-dependent apoptosis (Figure 4F)

**Figure 5:**
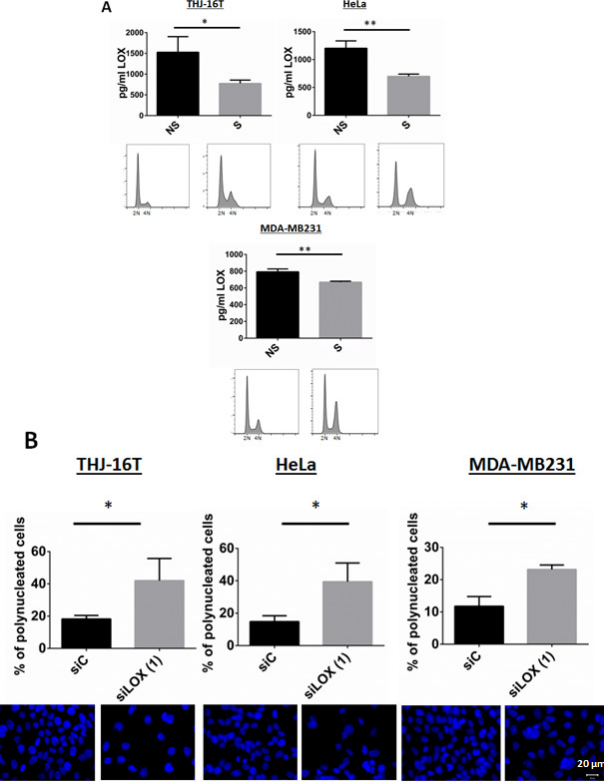
Secretion of LOX in synchronized cells (**A**) Released cells from a double thymidine block show a significant decrease in LOX secretion. A LOX ELISA test was used (see Materials and Methods). The bottom panel showed a G2/M arrest using PI staining after thymidine release. NS: Non-synchronized, S: Synchronized. (**B**) LOX knockdown increases the number of polynucleated cells 72 hr post-transfection. Top panel shows % of polynucleated cells counted. Bottom panel shows representative images with DAPI staining. *p < 0.05; **p < 0.01.

Since we observed that mitotic cells harbor a stronger staining of LOX, we asked whether the secretion of LOX is dependent on the phase of the cell cycle. The secretion of LOX was determined using an ELISA assay. Interphase cells were used as a control. LOX secretion in the medium was significantly reduced in mitotically synchronized cells (Figure 5A), suggesting that the binding of LOX to mitotic microtubules is associated with lower LOX secretion.

Because LOX interacts with the mitotic spindles, and loss of LOX increased the number of cells in G_2_/M phase and inhibited the expression of the major proteins involved in G_2_/M transition and mitosis, we next analyzed the morphology of LOX-silenced cells. LOX knockdown was associated with abnormally large and polynucleated cells as compared with control cells (Figure 5B). Taken together, these data showed that depletion of LOX, by inhibiting the major regulators of transition from G_2_ to M phase, leads to G_2_/M arrest and polyploidy, followed by apoptosis.

### LOX depleted cells are highly sensitive to anti-microtubule chemotherapeutic agents

**Figure 6:**
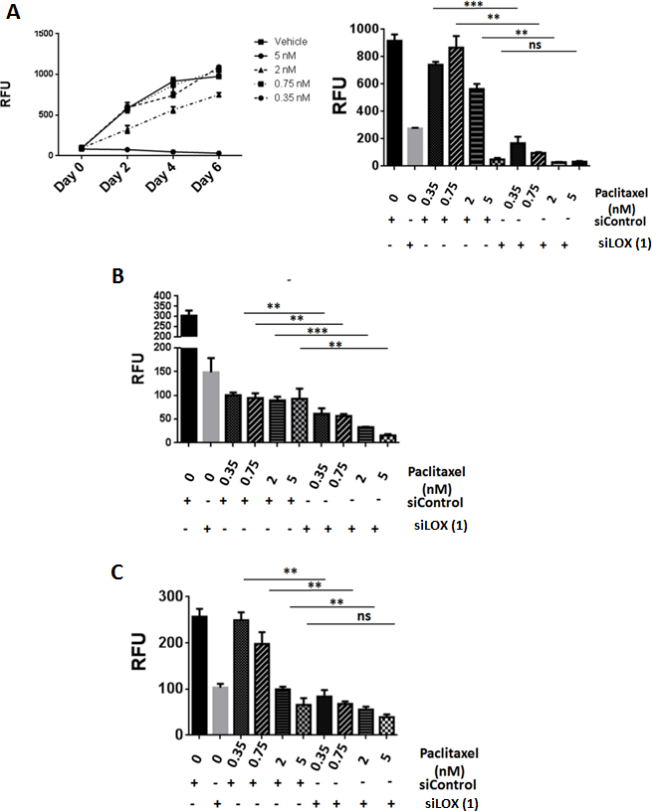
LOX knockdown enhances the effects of paclitaxel on cell proliferation (**A**) Left panel: Effects of increasing concentration of paclitaxel on cell growth in THJ-16T cells. Paclitaxel was combined with siLOX (1) or siControl in THJ-16T, MDA-MB231, and HeLa cells. Effect on cell number (day 4) in THJ-16T (right panel), HeLa (**B**), and MDA-MB-231 (**C**) cells. **P < 0.01, ***P < 0.001. RFU = Relative Fluorescent Unit. ns: non-significant.

**Figure 7:**
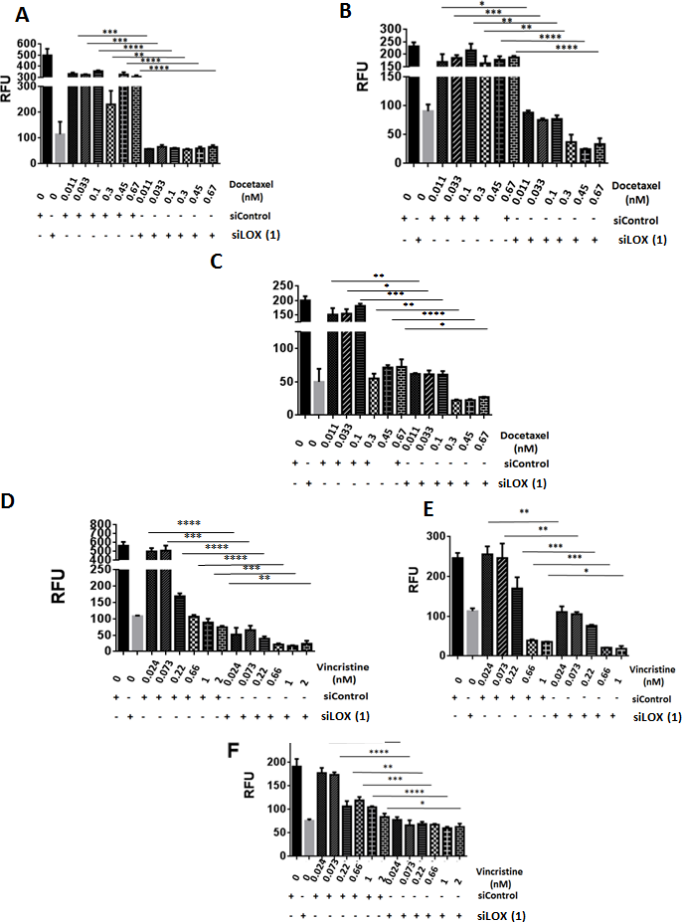
LOX knockdown enhances the effects of vincristine and docetaxel on cell proliferation (**A**, **B**, **C**) Growth inhibitory effects of Docetaxel as single agent or in combination with SiLOX (1) in THJ-16T (A), HeLa (B), and MDA-MB231 (C). (**D**, **E**, **F**) Growth inhibitory effects of Vincristine as a single agent or in combination with SiLOX in THJ-16T (D), HeLa (E), and MDA-MB231 (F). *P < 0.05, **P < 0.01, ***P < 0.001, ****p < 0.0001. RFU = relative fluorescent unit.

Since we found LOX to be associated with microtubules in mitotic cells, we postulated that LOX may be important in determining the sensitivity of cancer cells to chemotherapeutic agents that target microtubules. As expected, paclitaxel inhibited cellular proliferation in a dose-dependent fashion (Figure 6A, left panel). Interestingly, significantly increased cell death occurred in LOX-depleted cells treated with low concentrations of paclitaxel. These effects were even more pronounced after four days of paclitaxel treatment, which alone was unable to affect cell viability at low concentrations. Knockdown of *LOX* sensitizes THJ-16T, HeLa and MDA-MB231 cells to paclitaxel treatment (Figure 6A, right panel, 6B, and 6C). To confirm these results, we used two additional anti-microtubule agents, the Vinca Alkaloid vincristine and the Taxane docetaxel. Knockdown of LOX increased the anti-proliferative effect of docetaxel in the 3 cancer cells (Figure 7A, 7B, 7C), and increased the antiproliferative effect of vincristine in THJ-16T and HeLa cells (Figure 7D, 7E, 7F). These results indicate that inhibition of LOX expression potentiates the cytotoxic effects of anti-microtubules agents and these effects further support the role of LOX in cell cycle regulation, mitotic entry and its interaction with mitotic spindles.

## DISCUSSION

Because LOX has been associated with aggressive cancers and metastasis, it is important to characterize the intracellular functions of LOX. Previous to this work, we have shown that loss of LOX expression induces apoptosis in ATC cells. Here we have identified an unexpected role of LOX (a) in cell cycle regulation and (b) in cancer cell mitosis by directly interacting with microtubules.

In this study, we found that LOX is present in the cytoplasm during interphase, and then during mitosis, LOX is recruited to the mitotic spindles throughout mitosis, including spindle midzone during cytokinesis. We also characterized the effects of *LOX* gene silencing on cell morphology and cell cycle progression. Knockdown of LOX in cancer cells resulted in a progression through S phase with normal kinetics, followed by a significant increase of 4N cells. Several key proteins are required for proper progression of the cell cycle from G_2_ into M phase. In late G_2_ phase in mammalian cells, phosphorylation of H3 is first detected in pericentromeric heterochromatin; in late prophase, the phosphorylation is completed, and it is maintained through metaphase. Dephosphorylation of histone H3 begins in anaphase and ends during early telophase. In early G_2_ phase, AUKB is responsible for chromosome condensation by the phosphorylation of histone 3 on Ser10 [13]. During mitosis, AUKB plays a crucial role in the attachment between microtubules and kinetochores, and regulates the chromatids’ separation [17, 18]. Cyclin B1 expression is increased in G2 and maintained until metaphase, in which cyclin B1 has to be degraded before the cells progress through anaphase [23]. The cyclin B1/CDK1 complex is essential for the mitotic entry and progression from prometaphase to metaphase. Analysis of these key mitotic regulatory proteins showed that knockdown of LOX results in a decrease of cyclin B1, CDK1, and H3 phosphorylation at ser^10^ and AUKB. After binding to the cyclin-CDK complexes, p21 inhibits their kinase activities thereby preventing cell cycle progression [24]. Thus, the lack of LOX leads to an arrest in S phase and in G2 prior to mitotic entry. Studies have shown that when DNA is damaged, downregulation of DNA replication is observed [25]. G_2_/M Checkpoints are operational in late G2 to prevent damaged DNA in being segregated during mitosis [26]. Our results indicate that several essential markers of the G2/M checkpoint are regulated by LOX, and this may be reminiscent of replicative stress in cells deficient for LOX. Thus, it might be possible that LOX downregulation, by affecting the proper assembly of the mitotic spindle compromises the cell division, and this may further enhance the replicative stress, as elicited by CDK1/cyclinB1 regulation [27, 28]. Our data suggest that LOX is a key regulator of the transition from G2 to M phase. Arrest in G2/M phase can either lead to a senescence-like phenotype or to apoptosis [29]. Our study showed that a lack of LOX induces caspase-dependent apoptosis. Taken together, these findings suggest that the G_2_/M arrest observed with LOX knockdown explains, in part, the occurrence of apoptosis in LOX-depleted cells.

We found that LOX secretion was decreased during G_2_ and M phases. There have been several studies conducted to understand how the secretory pathway is altered during cell division. Given that protein synthesis is downregulated during mitosis to 25–30% of interphase levels, it is conceivable that the reduction in LOX protein secretion could be due to a general decline in protein synthesis [30]. However, confocal images showed strong LOX staining in mitotic cells, suggesting that the decrease in LOX in the cell culture supernatant of synchronized cells is not due to an inhibition of general protein secretion but due to its sequestration on mitotic spindles.

We provide evidence that LOX is recruited and interacts specifically with the mitotic microtubules, suggesting a function for LOX in spindle assembly and/or spindle orientation. The deleterious effects of LOX depletion compromise cellular proliferation and lead to gigantic nuclei. We believe that this is particularly significant because several studies have associated LOX with tumor aggressiveness, and our findings shed light on the mechanism behind this phenotypic observation.

Anti-microtubule agents have been used for cancer therapy for many decades. Although many of these agents that target the microtubules during cancer cell mitosis have shown good efficacy, they have been limited by off-target effects and toxicity when used at high concentrations or doses. We evaluated the effect of paclitaxel, docetaxel and vincristine and LOX knockdown alone and in combination. Our data show that LOX depletion sensitizes cancer cells to anti-microtubule agents. This finding provides further evidence that LOX interacts with microtubules during mitosis and can sensitize cancer cells to anti-microtubule agents. Thus, we expect that a strategy of reducing LOX expression in cancer cells could make existing cancer drugs that target the microtubules even more effective, with less toxicity as lower concentrations of the agent could be used.

The major components of the mitotic spindles are the microtubules that are assembled from heterodimers of α-tubulin and β-tubulin. Alpha and β tubulins have several lysine residues that are either exposed to the outside of the microtubule or at the interface of α-tubulin and-β tubulin [31]. Since LOX is responsible for the catalysis of collagen and elastin cross-linking within the extracellular matrix by catalyzing the exchange of an amine to an aldehyde group on a peptidyl lysine, LOX may interact with mitotic microtubules through their lysine residues. However, our data shows that the catalytic activity of LOX is not required for a G_2_/M transition and normal mitosis. Thus, the other protein domains of LOX may be involved in the interaction with mitotic spindles. Although our data reveal that LOX interacts with mitotic microtubules, suggesting a new function for LOX, it is still unknown whether this interaction is direct or indirect. A previous study performed in non-mitotic osteoblasts, has shown that LOX-propeptide is localized in the Golgi apparatus and active LOX can interact with tubulin, however, neither nocodazole nor paclitaxel modified this binding. These observations do not suggest any function of LOX in cell proliferation or interaction of LOX with mitotic spindles, unlike our data showing that LOX binds only to polymerized and stabilized microtubules with paclitaxel in mitotic cells [32].

In summary, our data provide the first evidence that LOX is involved in cell cycle regulation and is associated with mitotic spindles, indicating that inhibition of LOX is likely to be an effective anticancer strategy in cancers with high LOX expression, such as ATC, or breast or colon cancer, and in which microtubule inhibitors are in use for cancer therapy.

## MATERIALS AND METHODS

### Cell culture, transfection, and drug treatment

The THJ-16T anaplastic thyroid cancer cell line (with *TP53*, *RB*, and *PI3KCA* mutations) was kindly provided by Dr. John A. Copland (Mayo Clinic, Jacksonville, FL). The HeLa cell line was purchased from ATCC (Manassas, VA). The MDA-MB123 breast cancer cell line was provided by the cell repository of the National Cancer Institute (NCI, Frederick, MD). The cell lines were maintained in Dulbecco's Modified Eagle Medium (DMEM) with D-glucose (4,500 mg/L), L-glutamine (2 mM), and sodium pyruvate (110 mg/L), supplemented with 10% fetal calf serum (FCS), penicillin (10,000 U/mL), streptomycin (10,000 U/mL), and fungizone (250 mg/mL), all in a standard humidified incubator at 37°C, in a 5% CO_2_ atmosphere.

For the siRNA transfection, the cells were seeded in 6-well plates and transfected using Lipofectamine RNAiMax (Invitrogen, Thermo Fisher Scientific, Rockford, IL) with either siControl, siLOX (1) or siLOX (2), and harvested 72 hr after transfection.

For the overexpression of LOX, 250 000 HeLa cells were transfected with 0.75 ug of HIV-lentiviral–LOX vector (GeneCopeia, Rockville, MD) using Lipofectamin 3000 (Invitrogen, Thermo Ficher Scientifc). 36 hr after transfection the cells were washed and fixed for immunofluorescence staining.

For the LOX catalytic activity inhibition study, the cells were treated for 48 hr with 100 μM β-aminopropionitrile (BAPN, Sigma Aldrich, St. Louis, MO).

### Cell proliferation and colonies formation assay

For the cell proliferation assay, 1,500 cells were seeded and transfected in a 96-well plate. The CyQUANT Cell Proliferation Assay kit (Invitrogen, Thermo Fisher Scientific) was used to evaluate cell growth, according to the manufacturer's instruction. For the clonogenic assay, 1,000 cancer cells transfected with SiC, siLOX (1) or siLOX (2) were seeded in 6-well plates. After 10 days, the cells were washed with PBS and stained with 0.5% crystal violet.

### Flow cytometry

Cells were incubated with 10 μM BrdU for 2 hours prior harvest. Cells were then trypsinized and fixed overnight at 4°C in 70% ethanol in calcium- and magnesium-free phosphate buffered saline (PBS). Ethanol solution was removed and cells were incubated in 3 ml of 0.08% pepsin in 0.1 N HCl at 37°C for 20 minutes. Pepsin was removed and nuclei were incubated in 1.5 ml of 2 N HCl at 37°C for 20 minutes. The nuclei containing acid solution was neutralized with 3 ml of 0.1 M sodium borate. Nuclei were spun out of neutralized acid and washed with 2 ml IFA buffer (10 mM HEPES pH 7.4, 150 mM NaCl, 4% FBS and 0.1% sodium azide with 0.5% Tween-20 and then incubated overnight at 4°C with anti-BrdU clone MoBU-1 conjugated to AlexaFluor488 (Invitrogen B35130) in IFA buffer. DNA was stained for 30 minutes with the FxCycle containing propidium iodide (PI) RNase A (Life technologies, Thermo Fisher Scientific). Cell cycle analysis (bivariate plots of BrdU incorporation and DNA content) was performed on a FACSCanto II (Becton Dickinson, Franklin Lakes, New Jersey). Data were collected and analyzed using FlowJo software (FlowJo, Ashland, OR).

### Apoptosis assay

Caspase-Glo^®^ 3/7 substrate (Promega, Madison, WI) was added to 30,000 transfected cells and then incubated for 30 minutes at room temperature. Caspase activity was measured in each sample using a SpectraMax microplate reader (Molecular Devices, Sunnyvale, CA).

### Cell synchronization and mitotic spindles extraction

10. 10^6^ HeLa cells were treated with 10 μM nocodazole (NZ) for 6.5 hr, and the mitotic cells were harvested by shake-off and centrifuged for 2 min at 1,700 rpm. The pellets were washed with NZ-free medium, resuspended in 1ml, split into two equal portions in Eppendorf tubes and incubated at 37°C. Small aliquots of the suspensions were monitored under bright field microscope and when the re-formation of metaphase plates became evident (45 min), one of the mitotic cells suspensions was supplemented with NZ (10 μM final concentration) and the second one with paclitaxel (10 μM final). After another 20 min incubation at 37°C, the suspensions were centrifuged for 2 min at 200 g, the supernatants were discarded and the pellets reconstituted in 50 μl hypotonic, 10-times diluted General Tubulin Buffer (GTB, 1 × concentration 80 mM PIPES, pH 6.9, 1 mM EGTA and 1 mM MgCl_2_, pH 6.9) supplemented with Complete, EDTA-free, Protease Inhibitor Cocktail (Roche, Indianapolis, IN) and either 10 μM NZ or 10 μM paclitaxel. After 15 min at 37°C, the cells were lysed by the addition of 1% (final) Triton-X 100. The lysed cell suspensions were homogenized by pipetting and incubated for 2 min at room temperature. Approx. 10% of each suspension was fixed by mixing with 15-fold excess 4% PFA in GTB and allowed to adhere to poly-Lysine-coated coverslips (BD Biosciences) overnight before immunofluorescence staining. The remainder of each sample was centrifuged for 3 min at 16000 g. The supernatant and pellets were isolated and analyzed by Western blot using anti-tubulin (Cell Signaling) or anti-LOX antibody (Abcam).

### Western blot analysis

Cells were washed with PBS, and total cellular lysates were prepared by directly lysing the cells in the plates with 200 μl of lysis buffer (10 mM Tris-HCl, pH 7.4, 1% SDS). Protein concentration in the lysates was determined by BCA assay (Pierce, Thermo Fisher Scientific). Proteins were heated at 75°C for 10 min in the presence of LDS and Reducing agent (Invitrogen, Thermo Fisher Scientific), resolved by SDS-polyacrylamide gel electrophoresis in 4–12% SDS gels and transferred to PVDF (Invitrogen, Thermo Fisher Scientific) membranes by electroblotting. The membranes were then incubated overnight at 4°C with the following antibodies: LOX (1/1000, AbCam, Cambridge, MA); HEC1 (1/1000, AbCam); cyclin B1 (1/1000, Cell Signaling Technology, Beverly, MA); p-H3^(Ser10)^ (1/1000, Cell Signaling); AUKB (1/1000, Cell Signaling); p21 (1/500, Santa Cruz Antibodies, Santa Cruz, CA.) the blots were developed using ECL (Thermo Scientific, Life Technologies). For the mitotic spindles extraction, the pellets and the supernatants were mixed with lysis buffer, boiled and the protein concentration was measured. The samples were then reconstituted in bromphenol-blue and 5% β-mercaptoethanol-containing SDS PAGE sample buffer before electrophoresis and immunoblotting, using LOX (1/1000, AbCam); α- tubulin (1/1000, E7; DSHB Univ. of Iowa). The blots were developed with infrared dye-labeled secondary antibodies, using Odyssey Infrared Imaging System (LI-COR BioSciences, Lincoln, Nebraska).

### Immunofluorescence staining and confocal microscopy

Forty-eight hours after transfection with siControl or siLOX, 250,000 cells were plated in glass coverslips in 6-well plates and allowed to attach for 24 hr at 37°C and in a 5% CO_2_ atmosphere. Cells were then fixed in 4% paraformaldehyde (PFA) for 15 min. After permeabilization with 70% ethanol, cells were blocked with 5% BSA in PBS-TT (0.5% Tween and 0.1% Triton) for 1 hr at RT. The cells were immunostained for LOX using monoclonal antibody anti-LOX (Abcam), anti-tubulin (Cell Signaling Technology or DSHB Univ. Iowa), and anti-pH3^(Ser10)^ (Cell Signaling Technology). Then the cells were incubated with appropriate secondary antibodies conjugated with Alexa Fluor 488 or Alexa Fluor 569 for 1 hr (Life Technologies, Thermo Fisher Scientific). DNA was stained with DAPI (Vector Laboratories, Burlingame, CA). For the immunofluorescence staining of the mitotic fractions the samples were fixed in PFA 4%, washed and reconstituted in PBS before overnight incubation on poly-lysine coated cover slips (BD Biosciences) at 4°C. The samples were then stained with Hoechst 33342 (Sigma Aldrich) to detect DNA and with tubulin and LOX antibodies as described above. Fluorescence images were detected by confocal microscope NLO 710 Zeiss, and images were collected using Carl Zeiss Zen Software (Zeiss, Germany).

### Cell synchronization and LOX supernatant level

Before the cell cycle analysis, cells were synchronized using a double thymidine block protocol. In brief, 2 mM thymidine was added to culture plates at 60% confluence for 8 hr. Cells were released from the first block by washing and replacing with fresh medium supplemented with 10% FBS. After 9 hr, cells were again exposed to 2 mMthymidine, and released 9 hr later by washing and replacing with fresh medium supplemented with 0.5% FBS.

An ELISA was performed for LOX using the commercially available ELISA kits (Uscn Life Science Inc., Wuhan, China). The microtiter plate provided in this kit is pre-coated with an antibody specific to LOX. Standards and samples were then added to the plate wells with a biotin-conjugated antibody preparation specific for LOX. Avidin conjugated to horseradish peroxidase was added to each microplate well and incubated. The color change was measured by spectrophotometry at a wavelength of 450 nm. The concentration of LOX in the samples was then determined by comparing the optical density of the samples to the standard curve.

### Statistical analyses

Statistical analyses were performed using GraphPad Prism 5 software (GraphPad Software). Parametric data were analyzed using a two-tailed *t*-test. A *p* value of *p* < 0.05 was considered statistically significant. Data are presented as mean ± SD.

## SUPPLEMENTARY MATERIALS FIGURES


