# Supplementary material for: LOX is a novel mitotic spindle-associated protein essential for mitosis
Source: Oncotarget. 2016 Apr 7;7(20):29023–35. doi: 10.18632/oncotarget.8628 (PMC5045375; doi:10.18632/oncotarget.8628)
Supplement: Supplementary file 1 [file oncotarget-07-29023-s001.pdf]

LOX is a novel mitotic spindle-associated protein essential for mitosis

Supplementary Materials

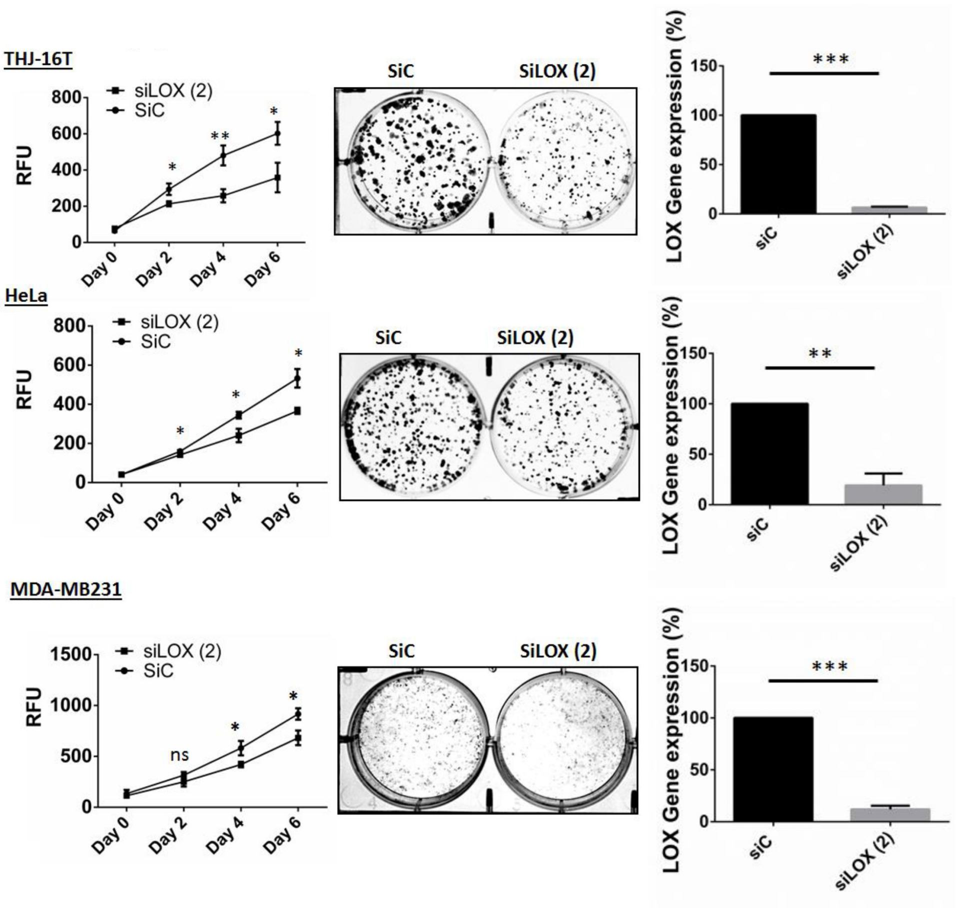

Supplementary Figure S1: Effects of siLOX (2) on cell proliferation. Knockdown of LOX using siLOX (2) inhibits LOX expression, cellular proliferation and colonies formation in THJ-16T, HeLa and MDA-MB231 cells.

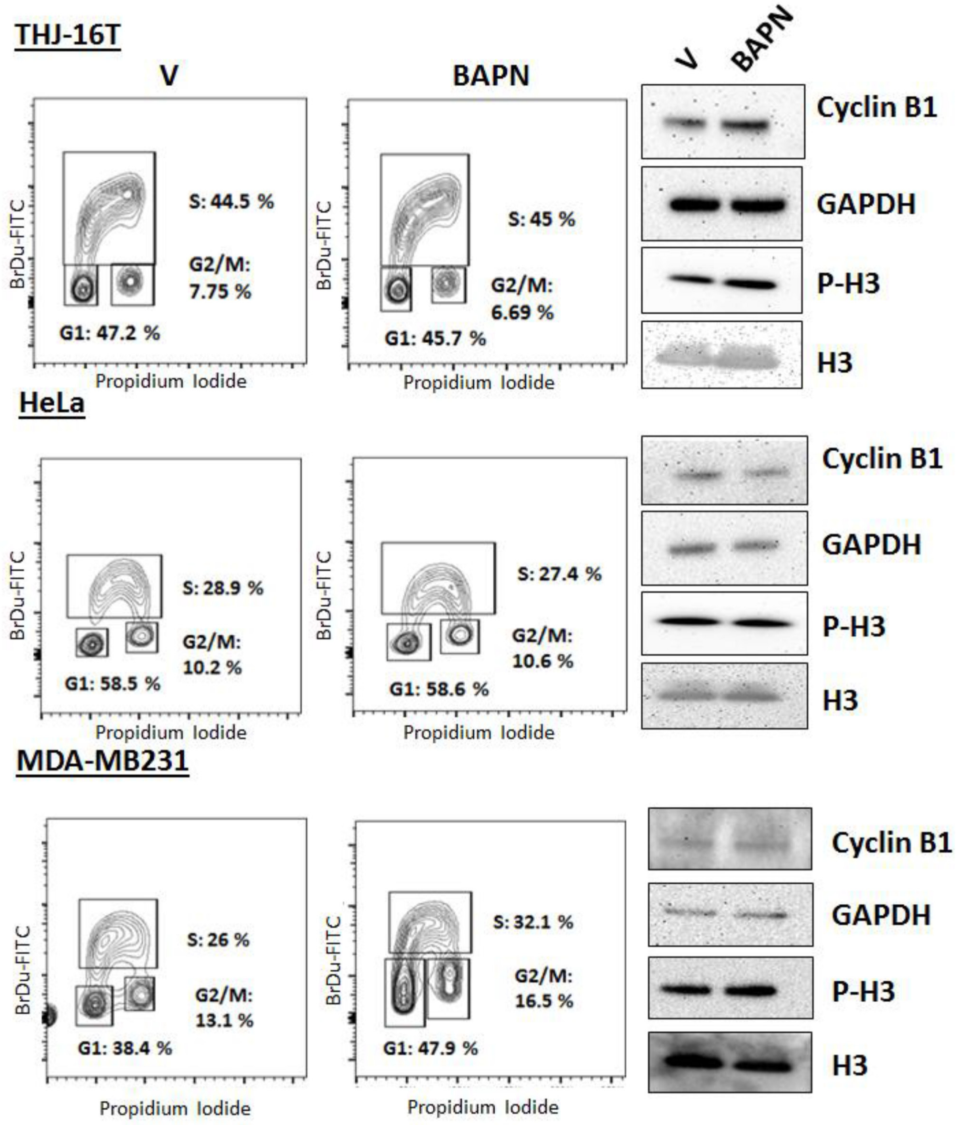

Supplementary Figure S2: Inhibition of LOX activity and cell cycle progression. Cell cycle progression and cell cycle regulators (p-H3$^{(Ser10)}$ and cyclin B1) were not inhibited by 48 hours of treatment with 100 μM of BAPN.
